# Giant ventral hernia—relationship between abdominal wall muscle strength and hernia area

**DOI:** 10.1186/s12893-016-0166-x

**Published:** 2016-08-02

**Authors:** K. Strigård, L. Clay, B. Stark, U. Gunnarsson, P. Falk

**Affiliations:** 1 Department of Surgical and Perioperative Sciences, Umeå University, Umeå, S-901 87 Sweden; 2 Department of Surgery, CLINTEC, Karolinska Institutet, Stockholm, S-171 64, Sweden, Karolinska University Hospital, Stockholm, S-171 64 Sweden; 3 Department of Molecular Medicine and Surgery, Karolinska Institutet and Department of plastic and reconstructive surgery, Karolinska University Hospital, Stockholm, S-171 64 Sweden; 4 Fibrinolysis Laboratory/Tissue Centre, Deptartment of Surgery, Institute of Clinical sciences, Sahlgrenska Academy, at University of Gothenburg, Sahlgrenska University Hospital/Ostra, Göteborg, S-416 85 Sweden

**Keywords:** Giant ventral hernia, Abdominal muscle strength, Hernia area, Biodex

## Abstract

**Background:**

Symptoms arising from giant ventral hernia have been considered to be related to weakening of the abdominal muscles. The aim of this study was to investigate the relationship between the area of the abdominal wall defect and abdominal wall muscle strength measured by the validated BioDex system together with a back/abdominal unit.

**Methods:**

Fifty-two patients with giant ventral hernia (>10 cm wide) underwent CT scan, clinical measurement of hernia size and BioDex measurement of muscle strength prior to surgery. The areas of the hernia derived from CT scan and from clinical measurement were compared with BioDex forces in the modalities extension, flexion and isometric contraction. The Spearman rank test was used to calculate correlations between area, BMI, gender, age, and muscle strength.

**Result:**

The hernia area calculated from clinical measurements correlated to abdominal muscle strength measured with the Biodex for all modalities (*p*-values 0.015–0.036), whereas no correlation was seen with the area calculated by CT scan. No relationship was seen between BMI, gender, age and the area of the hernia.

**Discussion:**

The inverse correlation between BioDex abdominal muscle strength and clinically assessed hernia area, seen in all modalities, was so robust that it seems safe to conclude that the area of the hernia is an important determinant of the degree of loss of abdominal muscle strength. Results using hernia area calculated from the CT scan showed no such correlation and this would seem to concur with the results from a previous study by our group on patients with abdominal rectus diastasis. In that study, defect size assessed clinically, but not that measured by CT scan, was in agreement with the size of the diastasis measured intra-operatively.

The point at which the area of a hernia begins to correlate with loss of abdominal wall muscle strength remains unknown since this study only included giant ventral hernias.

## Background

Giant ventral hernia may develop after an abdominal surgical procedure but may also arise spontaneously from, for example, an umbilical or epigastric hernia. Factors disposing towards the formation of hernia are postoperative infection, poor surgical technique, habitual factors such as smoking, and other disease such as diabetes, obesity and altered collagen metabolism [1, 2]. Alterations in extracellular matrix (ECM) formation and/or remodeling during wound healing are crucial in the development of abdominal wall defects indicating that proteases, cytokines and growth factors are key components of regulation [3]. Incisional hernia occurs in 10–23 % of patients after abdominal surgery and is even more frequent after emergency procedures [4].

Besides the complex biological and surgical burden, the patient has problems with clothing, personal hygiene and the sense of general weakness of the abdominal wall [5]. This, in turn, may lead to reduced physical activity resulting in obesity and a negative spiral is thus created. The considerable decrease in risk for recurrence, due to improved surgical technique and biomaterial used for reinforcement, has resulted in the need for new measures of outcome. Thus, Patient-Reported OutcoMeS (PROMS) and tools objectively evaluating functional outcome have become increasingly important. The surgical repair of hernia has been shown to result in considerable improvement in quality-of-life [6]. Since some patient complaints may be related to weak abdominal musculature, objective measurements are crucial if we are to evaluate the effects of hernia after laparotomy and the side effects of its repair.

It was shown in a small study that reduction of the Linea Alba in repair of ventral hernia increases abdominal wall muscle strength when measured using the BioDex system [7]. The BioDex is a complex dynamometer that may be used to evaluate the strength of the abdominal wall muscles [8]. BioDex has been used in sports medicine for several years and has been used in some studies evaluating abdominal wall muscle strength [8, 9]. Validation studies in persons without hernia and patients with giant hernia and rectus diastasis have been performed [10, 11]. Another method to estimate abdominal muscle strength is to count the number of straight and rotational curl-ups possible [12]. However, this method has not been widely used and has not been validated for this specific purpose. Furthermore, it is not possible to measure the strength of specific muscles in isolation using any of these methods. The abdominal girdle is complex and includes the rectus muscles as well as the external and internal abdominal oblique’s and transversus abdominis muscles. Any measurement of abdominal wall muscle strength represents the composite effect of these muscles. The techniques available for the evaluation of abdominal muscle strength were recently reviewed in a paper from Denmark [13].

We still do not know whether decrease in muscle strength of the abdominal wall correlates with the size of the ventral hernia. No study using objective measurements to compare hernia size to abdominal wall muscle strength has been published. The aim of this study was to determine whether or not giant ventral hernia area is correlated to decrease in abdominal wall muscle strength measured with the BioDex system.

## Methods

In a randomized controlled study on giant ventral hernia repair, preoperative hernia size and abdominal wall muscle strength were measured for all patients. A total of 52 patients scheduled for surgery for giant ventral incisional hernia were included. The hernias had a transverse size of at least 10 cm assessed either clinically or by CT scan. Exclusion criteria were <18 years of age, pregnancy, not able to understand written and verbal information, and smoking.

A CT scan was performed prior to surgery to exclude reasons other than giant ventral hernia for the patient’s abdominal discomfort, and for assistance in planning the surgical procedure. No Valsalva maneuver was performed during investigation.

The area of the hernia was calculated from clinical measurements (performed without Valsalva maneuver congruent to the measurements from CT-imaging) using a tape, and also independently assessed from CT images. Hernias were considered oval defects and therefore the formula for the area of an oval was used; the cranio-caudal and transverse dimensions were divided by 2 and the product of those multiplied by π. In the case of a “Swiss cheese” appearance, the outer borders were considered as the margin of the defect and the entire area was considered as one hernia defect.

Besides the area of the hernia, BMI, age and gender were also noted.

**Fig. 1:**
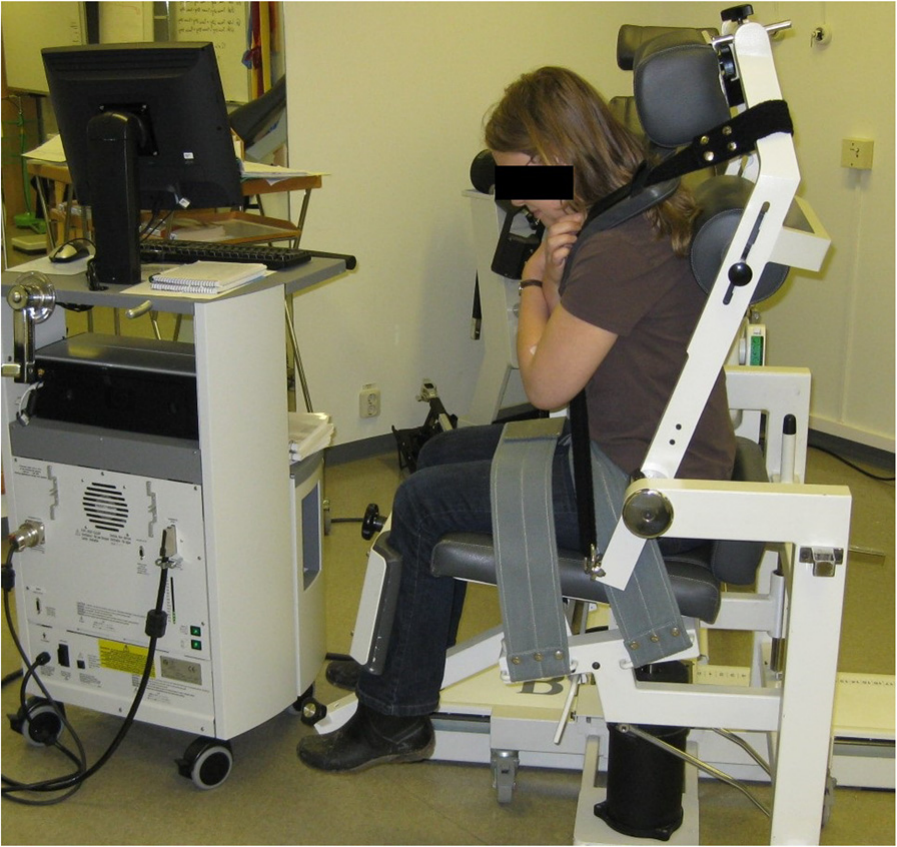
BioDex, System 4 with the back/abdominal unit. Member of the staff demonstrating Biodex. The patient is strapped in a fixed position. This prevents using other muscles than the abdominal corset and makes it possible to perform tests that are reproducible at a later stage

The BioDex System-4 together with a back/abdominal unit enables measurement of abdominal wall muscle strength. The modalities evaluated were flexion 30 and 60° per second, extension 30 and 60° per second, and isometric muscle contraction (Fig. 1). These tests were performed after instructions from one of two physiotherapists, and took 20 to 30 min to perform. Within each test there were five repetitions in rapid sequence, a short rest and then one further repetition of the entire set.

The study was approved by the Regional Ethical Review Board in Stockholm, 11/03/2009, reference number 2009/227-31/3. The research was performed in accordance with the Helsinki Declaration.

Written informed consent for participation was obtained prior to inclusion in the study.

### Statistical methods

Data were collected using an Access® database and calculated by STATISTICA version 12 (Statsoft, Tulsa, USA). The Spearman rank order test was used to compare the hernia area with the abdominal wall muscle force in each modality, and with BMI and age. The Mann-Whitney *U*-test was used to evaluate the influence of gender.

## Results

All 52 patients performed the BioDex test. There were 27 males and 25 females, median age was 47 years (range 34.8–77) and median BMI was 31.1 (range 22–46). In 15 cases the reason for the index operation resulting in the hernia was colorectal cancer or malignancy from testis, prostate, uterus, pancreas and gallbladder. Among the 37 benign conditions, 9 had had open bariatric surgery and 5 laparotomy due to diverticulitis. Thirty-three patients suffered from a cardiovascular disorder and 10 from diabetes.

**Fig. 2:**
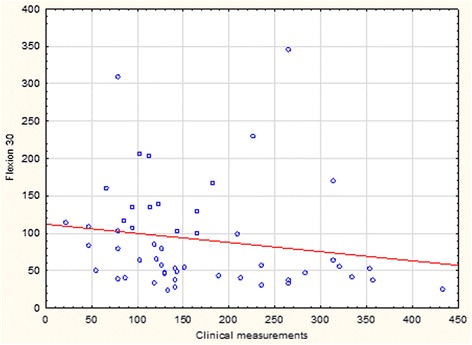
Scatterplot for 52 patients showing distribution of force in Nm generated at flexion 30^o^/s versus hernia area from clinical measurement in cm^2^

**Fig. 3:**
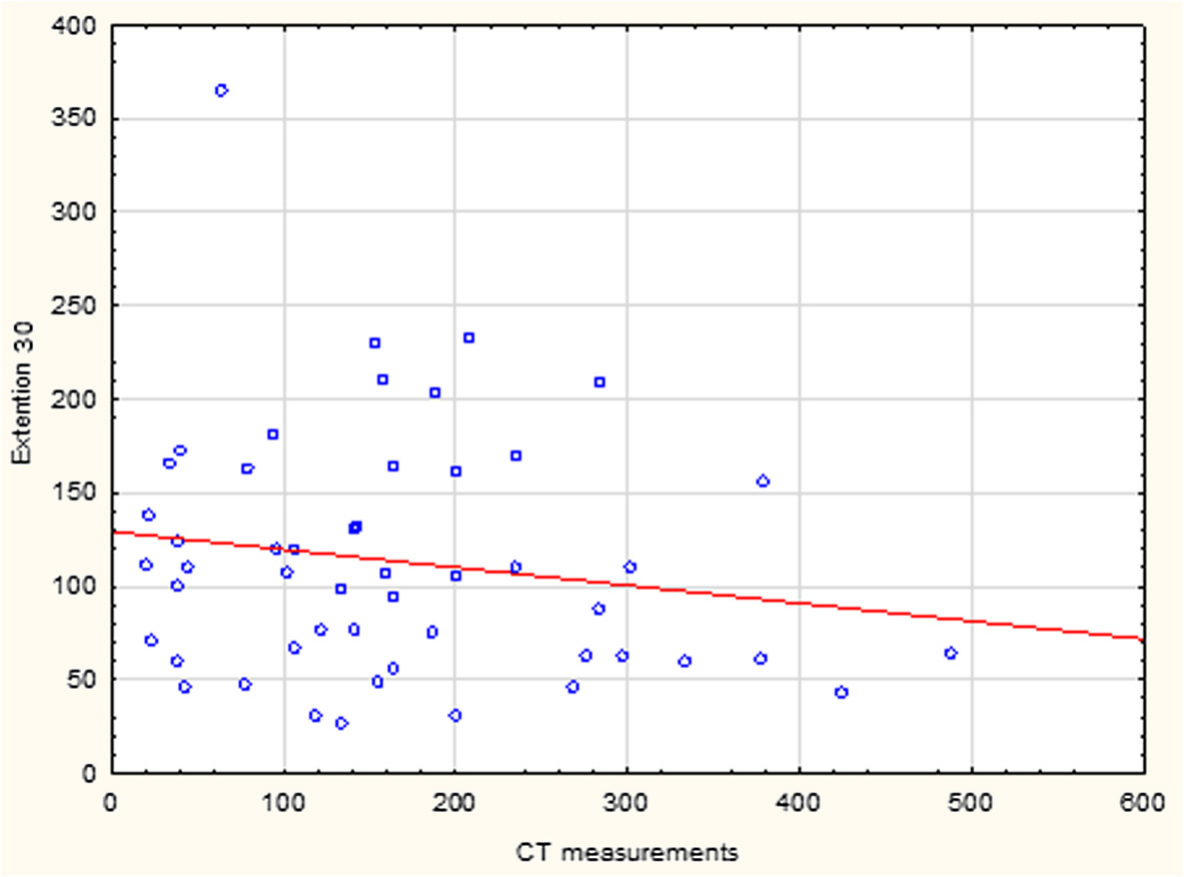
Scatterplot for 52 patients showing distribution of force in Nm generated at extension 30^o^/s versus hernia area from CT scan in cm^2^

The median area when calculated from clinical measurements was 137.4 cm^2^ (range 21.2–433.3). An inverse proportional relationship was revealed between force in all BioDex modalities and the clinically calculated area of the hernia. This inverse correlation was most pronounced for flexion and extension at 30° per second (Table 1). The force x area distribution is shown on a scatterplot for flexion at 30° per second (Fig. 2). Median hernia area using images from the CT scan was 148.0 cm^2^ (range 19.7–487.5). There was no correlation between the area calculated from the CT scan and any of the BioDex values in any modality (Table 2). The force x area distribution is shown on a scatterplot for extension at 30° per second (Fig. 3). Inter-patient variation in abdominal muscle force was wide, as seen in Table 3. The basic data set is available upon request from BMC Surgery.

**Table 1:** Spearman rank order correlation between hernia area derived from clinical measurement and BioDex force in flexion at 30 and 60^o^/s, extension at 30 and 60^o^/s and isometric contraction

Area derived from clinical measurements	n	Spearman R	t(N-2)	*p*-value
Flexion 30 ^o^/s	52	−0.33	−2.50	0.016
Flexion 60 ^o^/s	51	−0.30	−2.16	0.035
Extension 30 ^o^/s	52	−0.34	−2.53	0.015
Extension 60 ^o^/s	52	−0.31	−2.30	0.026
Isometric contraction	51	−0.32	−2.33	0.024

One patient failed to fulfill the program during flexion 60^o^/s and isometric contraction

**Table 2:** Spearman rank order correlation between hernia area derived from CT scan and BioDex force in flexion at 30 and 60^o^/s, extension at 30 and 60^o^/s and isometric contraction

Area derived from CT scan	n	Spearman R	t(N-2)	*p*-value
Flexion 30^o^/s	52	−0.15	−1.11	0.27
Flexion 60^o^/s	51	−0.03	−0.22	0.83
Extension 30^o^/s	52	−0.14	−1.02	0.31
Extension 60^o^/s	52	−0.07	−0.48	0.64
Isometric contraction	51	−0.22	−1.61	0.11

One patient failed to fulfill the program during flexion 60^o^/s and isometric contraction

**Table 3:** BioDex testing

BioDex Modality	Median (Nm)	Min (Nm)	Max (Nm)
Flexion 30^o^/s	54.9	23.6	345.6
Flexion 60^o^/s	78.8	37.2	294.9
Extension 30^o^/s	106.9	26.9	365.9
Extension 60^o^/s	108.9	35.6	281.9
Isometric contraction	50.5	14.2	200.2

Median, minimum and maximum forces in Nm recorded by the BioDex-4 system for each of the five modalities

There was no correlation between hernia area (calculated from clinical measurements) and gender, age (p ranging between 0.07 and 0.4 for the different modalities) or BMI (p ranging between 0.13 and 0.98 for the different modalities).

## Discussion

As the area of the hernia defect (measured clinically) increased, there was a proportional decrease in abdominal wall muscle strength (as Nm force). The abdominal wall muscle strength prior to the laparotomy resulting in the hernia was unknown. Even so, the correlation between hernia area measured clinically and decrease in abdominal muscle strength in all BioDex modalities is so robust that it seems safe to conclude that the area of the hernia is an important determinant for the degree of function lost. Indirectly, this also indicates those patients who will benefit most from surgery, and perhaps those who should not wait until their hernia defect progresses to a giant hernia. Measurement of hernia size is taken as either the length and /or width of the defect or the area. However, the area may be the most important predictor of clinical outcome. In this study, all BioDex forces measured during flexion, extension and isometric contraction, showed a strong inverse relationship to the hernia area determined from clinical measurements. One could have expected only the force of isometric contraction to be inversely proportional to the area since previous studies have shown that patients with a giant hernia have more pronounced difficulty in performing exercises involving movements of extension/flexion [10]. This may be due to a reduction in their capacity to perform repetitive movements as opposed to isometric contraction where there is a constant strain but no movement. On the contrary Criss et al showed improvement in both flexion and extension work load after abdominal hernia repair [7]. Criss also showed that all 13 of his patients showed an improvement in quality-of-life measured with HerQles, a validated questionnaire [14]. This is an important improvement and needs further investigation. It is difficult to design objective methods to determine the strength of the abdominal wall. Curl-ups have been tested [12] but this method is hard to reproduce. The BioDex system is probably the most precise and reproducible method to measure abdominal muscle strength, though only the combined strength of all muscles is measured [7, 8]. Testing could be further improved if EMG is added.

BMI, gender and age were not correlated to abdominal wall muscle strength. Thus no multivariate analyses, for example linear regression analysis, were performed.

At present it is not possible to determine which patients will benefit most from surgical hernia repair and many aspects must be considered before deciding on surgery. Important aspects include the risk for serious adverse effects emanating from synthetic reinforcement material, abdominal pain after surgery, and other risks related to anaesthesia and surgery [15]. It is thus of paramount importance to assess patient satisfaction using, for instance, a questionnaire as one PROM regarding pain related to function [14–16] as well as objective functional measurements such as muscle strength measured by the BioDex system before deciding on surgery.

It could be hypothesized that preoperative pain from a giant hernia may result in weak abdominal muscles and resulting back pain. These patients have difficulty in performing sports due to both a high BMI and spatial limitations from their giant hernia. It is well known that patients with inguinal hernia and pain before surgery are more prone to suffer from long-term postoperative pain. It is still not known if this is also true for giant ventral hernia, and this is an important field of research. At present there is only one study suggesting that patients with pain from ventral hernia are more prone to develop long-term pain after surgery [17]. If such a relationship is confirmed for giant ventral hernia, it would suggest that these patients should be operated as soon as the hernia causes the patient problems, not waiting until pain arises from weak abdominal muscle strength, or until incarceration or other imperative symptoms occur.

It is a matter of debate as to which ventral hernias require surgery, and when. Small hernias are usually repaired due to the high risk for incarceration of the intestine. In contrast, surgery for giant ventral hernia has a high complication rate including the risk for difficulty in breathing and disturbance of wound healing. Small hernias, less than 15 mm, may be sutured without the application of a mesh, while giant hernia repair requires some form of reinforcement [1, 18]. den Hartog showed improved muscle strength after suture repair compared to laparoscopic repair with mesh, but the size of the hernias was not given [9]. It can also be hypothesized that some hernia patients may have impaired muscle strength due to an imbalance in ECM formation or remodeling capacity, resulting in weaker collagen and stroma structures, a status that also predisposes to hernia. Several factors are involved in ECM remodeling including a series of zinc- dependent matrix metalloproteinases (MMPs). Antoniou et al demonstrated in 2011 that local tissue levels of MMP-2 and -9 were increased while systemic levels of the same MMPs were decreased in most patients with inguinal hernia [19]. Moreover, an imbalance between collagen I/III and MMP 1 has been shown to predispose to hernia [4]. It is possible that such patients are more likely to benefit from surgery, and that monitoring the levels of both local and circulating MMPs may improve patient selection. However, this remains to be seen.

Hernia patients often receive contradictory advice regarding physical activity. In the case of giant ventral hernia it is still an open question whether exercise of the abdominal wall muscles leads to greater diastasis or whether it is possible to increase muscle mass thereby bringing these muscles together. In patients suffering from diastasis recti it has been shown that the width of the Linea Alba can be reduced by exercise [20]. When including active training in the work-up prior to surgery, close collaboration with an involved physiotherapist is important to avoid any side effects of muscle exercise.

An interesting observation in the present study was that the hernia area determined by CT scan showed no correlation to abdominal muscle strength. Width of abdominal rectus diastasis by CT scan has previously been shown not to correspond to that measured clinically, the latter more closely representing the “truth” found at surgery [21]. One could speculate that muscle fibers identified by the CT scan represent all fibers, regardless of whether they are functional or largely consist of fibrous tissue. Clinical evaluation, on the other hand, probably only takes into account muscles with the capacity to contract on demand. Should this be the case, the lack of correlation between hernia area measured by CT scan and abdominal muscle strength, seen in this study, would seem logical. There was no evaluation of inter-rater reliability included in the present study; the length and width of the hernia was requested at the original radiological investigation on admission. A previous study has shown a high degree of inter-rater reliability when determining the width of the diastasis between the rectus muscles from CT scans [20]. CT scanning, however, remains an essential investigation in the preoperative assessment of patients suffering from a ventral hernia; not for an exact evaluation of the size of the abdominal wall defect, but to detect associated pathology and for assistance in the planning of the surgical procedure.

A large prospective study measuring preoperative abdominal wall muscle strength is required to see if patients who develop a giant hernia also had weaker abdominal muscles prior to the index abdominal operation causing the hernia. Furthermore, a study on local MMP levels in muscle and fascia biopsies as well as systemic levels, in patients with a hernia may give more insight into the pathogenesis of this disorder.

## Conclusion

This study of patients with a giant ventral hernia reveals an inverse proportional relationship between the hernia area and abdominal wall muscle strength. The point at which this relationship begins to apply still remains to be determined since the present study included only cases with a transverse diameter exceeding 10 cm, either by clinical measurement or CT scan.

## Abbreviations

BMI, body mass index; Cm, centimeter; CT, computed tomography; EMG, electromyography
